# Computation of Gait Parameters in Post Stroke and Parkinson’s Disease: A Comparative Study Using RGB-D Sensors and Optoelectronic Systems

**DOI:** 10.3390/s22030824

**Published:** 2022-01-21

**Authors:** Veronica Cimolin, Luca Vismara, Claudia Ferraris, Gianluca Amprimo, Giuseppe Pettiti, Roberto Lopez, Manuela Galli, Riccardo Cremascoli, Serena Sinagra, Alessandro Mauro, Lorenzo Priano

**Affiliations:** 1 Department of Electronics, Information and Bioengineering, Politecnico di Milano, Piazza Leonardo da Vinci 32, 20133 Milano, Italy; veronica.cimolin@polimi.it (V.C.); roberto.lopez@biomedica.udec.cl (R.L.); manuela.galli@polimi.it (M.G.); 2 Istituto Auxologico Italiano, IRCCS, Department of Neurology and Neurorehabilitation, S. Giuseppe Hospital, 28824 Piancavallo, Italy; lucavisma@hotmail.com (L.V.); riccardo.cremascoli@unito.it (R.C.); serenasinagra@gmail.com (S.S.); alessandro.mauro@unito.it (A.M.); 3 Department of Neurosciences, University of Turin, Via Cherasco 15, 10100 Torino, Italy; 4 Institute of Electronics, Computer and Telecommunication Engineering, National Research Council, Corso Duca degli Abruzzi 24, 10129 Torino, Italy; claudia.ferraris@ieiit.cnr.it (C.F.); gianluca.amprimo@ieiit.cnr.it (G.A.); giuseppe.pettiti@ieiit.cnr.it (G.P.); 5 Department of Control and Computer Engineering, Politecnico di Torino, Corso Duca degli Abruzzi 24, 10129 Torino, Italy; 6 Department of Electrical Engineering, Universidad de Concepción, Víctor Lamas 1290, Concepción 4030000, Chile

**Keywords:** RGB-D sensors, optoelectronic system, movement analysis, gait, Parkinson’s disease, hemiparesis, spatio-temporal parameters

## Abstract

The accurate and reliable assessment of gait parameters is assuming an important role, especially in the perspective of designing new therapeutic and rehabilitation strategies for the remote follow-up of people affected by disabling neurological diseases, including Parkinson’s disease and post-stroke injuries, in particular considering how gait represents a fundamental motor activity for the autonomy, domestic or otherwise, and the health of neurological patients. To this end, the study presents an easy-to-use and non-invasive solution, based on a single RGB-D sensor, to estimate specific features of gait patterns on a reduced walking path compatible with the available spaces in domestic settings. Traditional spatio-temporal parameters and features linked to dynamic instability during walking are estimated on a cohort of ten parkinsonian and eleven post-stroke subjects using a custom-written software that works on the result of a body-tracking algorithm. Then, they are compared with the “gold standard” 3D instrumented gait analysis system. The statistical analysis confirms no statistical difference between the two systems. Data also indicate that the RGB-D system is able to estimate features of gait patterns in pathological individuals and differences between them in line with other studies. Although they are preliminary, the results suggest that this solution could be clinically helpful in evolutionary disease monitoring, especially in domestic and unsupervised environments where traditional gait analysis is not usable.

## 1. Introduction

The world population is aging rapidly as a consequence of the longer life expectancy. According to an OECD analysis, by the middle of the 21st century, more than 20% of the world population will be over 65, and this demographic change will affect both industrialized and developing countries [1]. In addition, the World Health Organization estimates an increase in the neurological diseases linked to the aging trend, particularly those ones characterized by chronic or progressive disabilities such as Parkinson’s disease and stroke, with a consequent exponential growth of healthcare costs [2]. It is, therefore, possible to understand the importance of therapeutic assistance interventions which, in addition to improving the psycho-physical conditions of the individual, can reduce the costs of health care.

Stroke is one of the principal causes of morbidity and mortality in adults and one of the leading causes of disability in industrialized countries [3,4]. Hemiplegia/hemiparesis following stroke is one of the consequences of the acute loss of focal brain functions and it is clinically characterized by a deficit of voluntary motor activity in one half of the body, contralateral to the ictal lesion involving the primary motor cortex [5]. Six months after the event, 50–70% of patients still present sensory and motor deficits, such as paresis and spasticity of the limbs, which affect the ability to perform functional tasks, lead to reduced quality of life, and reduce participation in activities of daily living. The chronic motor disability then imposes significant challenges for prolonged treatment and patient care [6]. Focusing on post-stroke with hemiparesis condition, patients’ gait can be variably altered by the impairments of motor functions (weakness and spasticity of lower limbs) and by alterations in posture and balance controls [7]. 

Parkinson’s disease (PD) is the second most common neurodegenerative disorder, with a prevalence that increases with age [8]. The main characteristic is the progressive worsening of motor control and coordination capabilities induced by the death of dopaminergic neurons [9]. The expression of motor dysfunctions varies among subjects and over time. Nevertheless, subjects mainly exhibit some typical symptoms, including tremor, bradykinesia (slowness of movements), rigidity, postural instability and abnormalities, gait disorders, alterations in speech, mimicry, and writing [10]. As for stroke, the progression of motor disabilities imposes significant efforts for prolonged patient care and rehabilitation programs aiming to control symptom severity and enhance muscle strength, balance, gait, and mobility [11]. Focusing on gait, the alterations in walking patterns such as reduced speed, reduced step length, and increased step variability, combined with postural instability, lead to limited mobility and increased falling risk [12]. 

As gait impairments are experienced to be particularly disabling by patients, the walking recovery is a major objective in stroke and PD rehabilitation programs. Therefore, in the last decades, walking capability has been the object of study for the development of gait analysis methods in stroke survivors [13] and subjects affected by PD [14], both for diagnostic and rehabilitation purposes.

In the literature, the gait pattern in post-stroke subjects was quantitatively described using three-dimensional instrumented gait analysis (3D-GA), evidencing walking speed reduction, asymmetric postural behavior during walking and standing [15], and altered kinematics and reduced ankle push-off ability during terminal stance [16]. 3D-GA was also used in PD subjects to quantify the efficacy of rehabilitation [17] and to estimate spatio-temporal parameters [18,19]. 

3D-GA is widely used in clinical practice and research to investigate gait disorders, as it provides complete and objective information regarding specific gait features, including joint motion (kinematics), time–distance variables (spatio-temporal data), and joint moments and powers (kinetics). Conventionally, body segment kinematic and kinetic parameters are measured in gait laboratories, using marker-based optoelectronic systems and force plates. 3D-GA is considered accurate [20], but the availability of specific laboratories, high costs, and dependency on trained users [21,22] limit its use in clinical practice. Additionally, optoelectronic 3D-GA generally requires few clothes to be worn, and this condition could cause anxiety and embarrassment to patients [21,23].

Over the last decade, low-cost optical body-tracking sensors (i.e., RGB-D cameras) have been introduced in the gaming market as innovative devices for a new paradigm of human–computer interaction based on body movement. In particular, Microsoft Kinect© was the first device developed and released for this purpose. Although RGB-D cameras have not achieved significant success in the gaming market, they have subsequently found widespread use in other contexts, such as developing new rehabilitation systems for gait analysis, limb motion tracking, and gesture and posture classification [24,25]. Kinect-based solutions have become progressively more used in computer-assisted medical care and treatment thanks to lower costs and non-invasive motion capture techniques. Consequently, clinicians gained the possibility to explore the fields of body recognition and analysis of motor function as promising tools to monitor patients also in out-of-hospital settings. This need is considered extremely important for long-term care, for National Health System (NHS) costs, and the hospital service management and resource policies.

In recent years, several studies considered the use of RGB-D cameras for the assessment of the motor condition [26,27], for action and activity recognition [28], and motor rehabilitation [29], especially combined with virtual environments [30,31]. Regarding gait analysis [32], the first model of the Kinect sensor was widely used to analyze gait patterns in young adults [33,34], healthy adults [33,35], and children [36]. The characteristics of the next model include improvements of the on-board sensors, a wider field of view, stability of the body-tracking algorithm, and higher resolution of color and depth streaming, leading to higher performance and greater accuracy of motion capture than the older model [26,37,38,39]: the enhanced features allowed more detailed investigations of gait characteristics in healthy and pathological subjects. As for traditional gait analysis, some studies implemented a multi-camera-based system in order to cover greater walking distances (generally up to 10 m). However, this entails an increasing complexity of the implemented solution for the management, flow synchronization, and calibration procedures [40,41]. Other studies combined optical sensors and treadmills as an alternative approach to capture a more significant number of steps for the gait assessment [42,43]. On the other hand, the high cost and size of these approaches must be considered, which means that their implementation and use are still limited to motion analysis laboratories, such as for 3D-GA. Thus, conducting a walking test using these approaches could become impractical in smaller and unsupervised environments such as domestic settings. 

A more practical solution is to use a single camera approach, which makes it possible to carry out walking tests in more confined spaces. Solutions based on a single camera could be easily used in any environment and adapted to specific needs, thus addressing a relevant clinical requirement, that is, the availability of a valid and straightforward method for quantifying gait patterns in pathological subjects that is suitable for home settings. 

Single-camera approaches have been used to evaluate gait patterns in people with different health conditions, including cerebral palsy [44], ataxia [45], Parkinson’s disease [46], and polyneuropathy [47], or to analyze young and older individuals [48,49]. More generally, RGB-D sensors (i.e., Kinect-like optical sensors) have been used in PD subjects to evaluate upper limb tasks [50], lower limb and posture [51], and gait and postural stability [46]. Concerning post-stroke, RGB-D sensors have been used to predict the risk of falls [52], evaluate the upper limb function [53], analyze balance recovery [54], rehabilitate upper limbs [55], estimate gait features [56], and evaluate the reliability of gait assessment and correlation with balance tests [57]. However, validating the accuracy of these non-invasive devices in capturing gait outcomes appears to be mandatory. 

Studies on Parkinson’s Disease [58,59], individuals with hemiplegia and stroke [56,60,61], subjects with other pathologies and disorders [62,63,64,65], and healthy people [35,48] indicate that some spatio-temporal gait parameters could be appropriately estimated using a single-camera approach. Concerning the gait validation, some studies are available using treadmills [43,66,67], multi-camera approaches [40,41], or wearable sensors [12,68,69,70]. However, there is scarce information for comparing the spatio-temporal gait parameters estimated by a single RGB-D sensor and an optoelectronic system on PD subjects [46,71], post-stroke individuals [61], and those with both pathological conditions [60], especially through a solution that is suitable for unsupervised or semi-supervised environments. In fact, from a clinical point of view and to ensure the sustainability of the health system, it is essential to have a simple method to quantify gait strategies in these pathological subjects. However, at the same time, it is necessary to define the level of accuracy and any limitations to allow clinicians to target its use properly; for example, for diagnostic or rehabilitation purposes.

Along this line of research, this study aims to extend our previous work [72] by validating the spatio-temporal gait parameters in level walking for both post-stroke individuals and subjects with Parkinson’s disease. A single-camera approach on a reduced walking path, which is thus suitable for the limited spaces in domestic environments, is proposed to this end. The objective measurements estimated with this solution are compared with the corresponding ones obtained from the instrumented 3D-GA and other validation studies available in the literature, albeit on inhomogeneous subsets of spatio-temporal parameters, correlation criteria, and populations. This solution could become, for example, a valid method for evaluating and monitoring walking strategies over time, also integrating into more complex solutions [51] that assess the patient’s general condition frequently and in unsupervised environments, enabling the follow-up of patients even outside of healthcare facilities. The possibility of home monitoring would allow the clinicians to constantly assess the evolution of the disease and to target interventions according to real and current patient needs, with the patient being able to feel “cured” and the national health care system being able to save money through the careful management of hospitalization periods, when required, and personalized rehabilitation programs in both supervised and unsupervised settings. 

Furthermore, the non-invasiveness and versatility to different clinical needs could also favor an increasingly important role in future ecological neuro-rehabilitative contexts by stimulating patients at home and in daily activity [73,74] and ensuring the continuity of those services currently provided only from health facilities. 

In conclusion, the main goals of this study are summarized as follows: (i): evaluate the agreement and robustness of the estimated gait parameters obtained from a single-camera approach based on Microsoft Kinect v.2 versus an instrumented standard 3D-GA system; (ii): objectively characterize the gait patterns on a cohort of parkinsonian and post-stroke subjects; (iii): compare the estimated gait parameters with several studies in the literature.

## 2. Materials and Methods

In this section, the study design is described. Since the study aimed to characterize the gait patterns in pathological subjects using a technological solution suitable for the home setting, a validation phase was necessary to check accuracy and robustness versus an instrumented 3D-GA system. To this end, two groups of subjects were enrolled to participate in the experimental study. Then, a dedicated setup and an acquisition protocol were defined to allow for the simultaneous motion capture and the estimation of gait parameters through the two systems. Finally, statistical analysis was performed to compare parameters and evaluate the correlation, reliability, and significant differences between the two systems and the pathological groups. 

### 2.1. Patients

The recruitment procedure of PD and post-stroke participants to the experimental campaign, planned for this study and performed in a supervised setting, considered the potential end-users of the proposed system in home settings. In particular, we included only post-stroke (PS) and PD subjects who had already been evaluated and selected for standard rehabilitation in the hospital setting, and furthermore, who would have benefited from continuous and prolonged neurorehabilitation at home. Enrolment was then performed at the Division of Neurology and Neurorehabilitation, San Giuseppe Hospital, Istituto Auxologico Italiano, Piancavallo (Verbania), Italy. The inclusion criteria for post-stroke subjects were: minor disability of the lower limbs (possibility of walking), ability to walk 10 m without the assistance of another person or aids, ability to understand the instructions for performing the gait analysis test. The inclusion criteria for PD subjects were: tremor severity <=1, Hohen and Yahr (H&Y) score in 1–3 range. The exclusion criteria for both groups were: cognitive impairment with Mini-Mental State Examination (MMSE) < 27/30, previous neurosurgical procedures, history of other neurological or musculoskeletal disorders unrelated to stroke and PD. The exclusion criteria did not include age, sex, side dominance, or therapy. 

For the experimental test, we recruited eleven post-stroke subjects who presented partial anterior circulation infarcts (PACI) according to Bamford’s classification [75], and ten PD subjects who satisfied the inclusion criteria. 

The clinical supervisor explained the experimental procedure in detail and instructed all participants accurately about the systems and the acquisition protocol, so all participants performed the walking test under the same conditions. The experimental campaign was carried out following the ethical standards of Istituto Auxologico Italiano, whose local ethical committee approved the study, and the latest amendments of the Helsinki declaration (1964). The enrollment procedure required all participants to sign informed consent forms to participate in the study.

### 2.2. Characteristics of the RGB-D and 3D-GA Systems

A single RGB-D sensor was used to implement a non-invasive motion capture system that includes hardware and software elements. The hardware relies on the Microsoft Kinect© v2 sensor (Microsoft Corporation, Redmond, WA, USA) and an elaboration unit consisting of a laptop running Windows 10 to which the RGB-D sensor connects through a dedicated USB port. The optical sensor produces color and depth streams at about 30 frame/s, using the time-of-flight technology to estimate the depth information: these features are adequate for the real-time motion capture and 3D reconstruction of the human body movement. Human body movements map on a skeletal model consisting of 25 joints that approximately correspond to specific anatomical points of the body: the tracking algorithm recognizes body patterns via the depth streaming and identifies the spatial regions associated with the joints of the skeletal model through a random forest classifier trained on thousands of images [76]. For each joint, the relative 3D position to the origin of the sensor reference system is available and returned for the 3D reconstruction of the body movements. In our previous studies [51,72], the 3D trajectories of joints and segments were compared to a gold reference system, verifying the accuracy and robustness of angular and linear measurements.

The RGB-D motion capture system includes custom-written software consisting of MATLAB^®^ scripts (Mathworks Inc, Natick, MA, USA) that runs on the elaboration unit. The software component implements access, saving, and analysis procedures of the raw information provided by the RGB-D sensor through the Software Development Kit (SDK), including color images, depth images, and structured skeletal model data. The analysis procedure works on the collected 3D trajectories of joints to automatically segment every step and estimate the gait parameters that will be compared with the 3D-GA system: the Data Processing section (Section 2.4) describes the analysis procedure in detail. In addition, the RGB-D system has a Graphical User Interface (GUI), managed by MATLAB scripts, to simplify motion capture through specific utilities to check device operation, start and stop acquisition, and check data acquisition correctness [72]. 

To satisfy the validation aims, the study compared the RGB-D motion capture system to a standard 3D-GA system consisting of an optoelectronic system with six cameras (VICON, Oxford Metrics Ltd., Oxford, UK; sample rate: 50 Hz) and two force platforms (Kistler, Einterthur, CH). Optoelectronic systems represent the gold standard of technologies used in motion analysis for the evaluation of kinematics. After taking some anthropometric measurements, the operator placed the passive markers on the subject’s skin at specific key points [77]. The reference systems for each segment of the lower limbs were calculated starting from the 3D coordinates of the markers positioned on the pelvis, thigh, leg, and foot: the angles of flexion–extension, abdominal-adduction and intra–extra rotation of the joints of the lower limbs were computed. The kinematic (angles) and kinetic (moments and powers) data from the 3D-GA system were not used for this study. 

Before starting the experimental test, the 3D-GA system was calibrated to ensure the system’s accuracy and to allow the estimation of the 3D marker coordinates. The average measurement error was computed based on the difference between the estimated and actual distances of two passive markers fixed on the extremities of a rigid bar (actual distance was 600 mm): the calibration procedure ended with an average error within 0.3 mm (standard deviation: 0.2 mm). In this condition, the calibrated working volume was 5 m in length (*x*-axis of the laboratory reference system), 2 m in height (*y*-axis of the laboratory reference system), and 2 m along the *z*-axis of the laboratory reference system.

### 2.3. Setup and Data Acquisition

In the movement analysis laboratory of the Institute, traditional gait analysis was performed using a 10-m long walkway. However, considering that the body tracking of the RGB-D system begins approximately 4.5 m from the sensor, it was necessary to define a setup that allowed for a peer comparison between the two systems. The RGB-D sensor was mounted on a tripod to ensure stability and placed at the end of the walkway. In this way, it was possible to capture the participants’ gait as they moved towards the optical sensor that was preferable [41]. 

**Figure 1:**
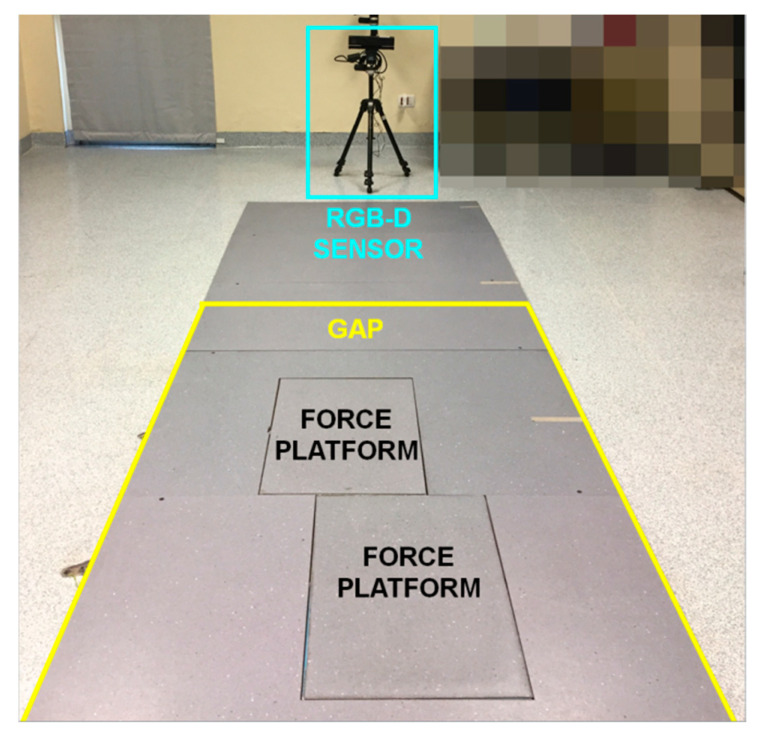
Experimental setup: position of the RGB-D sensor (light blue square); position of the force platform for 3D-GA system; approximate position of the final area of the Gait Analysis Path on the walkway for RGB-D system (yellow lines).

Then, a gait analysis path (GAP) was defined inside the walkway to ensure the total body tracking with the necessary accuracy: the start line was 4.2 m away from the sensor’s position, while the end line was about 1.5 m away. This setup seemed to limit the analysis to a restricted area of the walkway, which was, in any case, sufficient to capture at least one complete gait cycle (or full stride) for each leg based on the motor conditions of the participants [57]. Moreover, GAP was adequate to guarantee the maximum depth accuracy on which the body-tracking accuracy and robustness also depend: according to [78], the vertical and lateral scattering of light pulses affect the depth accuracy in the working volume. The greater precision was along the central visual cone of the RGB-D system, where the depth accuracy was less than 2 mm, increasing to 4 mm up to 3.5 m and over 4 mm beyond 4.0 m from the sensor. It was also necessary to consider that the GAP was set approximately across the walkway mid-zone and around the force platforms, thus enabling the simultaneous gait analysis using the 3D-GA system. Figure 1 shows the experimental setup. Particular attention was paid to avoiding the presence, on the scene, of reflective surfaces and light sources entering the RGB-D sensor: these elements could interfere with the light pulses emitted by the device, generating artifacts in the depth map and, consequently, errors in the detection of the body map and the reconstruction of the 3D skeletal model. These measures can become constraints, especially for domestic environments. In this scenario, further constraints concerned clothing: in particular, baggy and too dark clothing and reflective objects needed to be avoided as they could interfere with the accurate motion capture. All these requirements will be part of the domestic experimental protocol, and all participants will have to be trained and supported in the system’s configuration.

The acquisition protocol required participants to have completed two or more practice trials across the walkway to understand and be comfortable with the experimental procedure. After an initial familiarization, participants had to stand up and maintain an upright posture for a few seconds and then walk straight at their normal walking pace, from the beginning of the walkway to the RGB-D sensor. In this way, each subject entered and then left the GAP at his/her maximum walking ability. Only forward walking was considered for the gait analysis [79]. According to the experimental protocol, at least five trials were performed by each participant to guarantee the reproducibility of the results. 

### 2.4. Data Processing and Estimation of Gait Parameters

For the analysis of 3D-GA data, the passive markers were used to identify the main walking events from which to estimate spatio-temporal parameters. In particular, the first heel-strike event was detected for each foot from the force platforms. Then, the previous and following similar events were identified by comparing the first heel-strike kinematic configuration (consisting of foot position, hip/knee/ankle flexion angles, and other specific features) over the 3D-GA data collected during the entire walking test. These events allowed the detection of at least two or three complete gait cycles inside the GAP, according to the walking ability and health condition of the subject, which were the same as those recognized by the RGB-D system.

For the data collected by the RGB-D system, the analysis procedure used dedicated MATLAB scripts that worked on the 3D joints recorded during the walking test. The analysis procedure consisted of three phases: data preprocessing, step segmentation, and estimation of gait features. The preprocessing phase applied resampling and filtering techniques to clean and align the data to the 3D-GA ones before using them for the step segmentation phase. In particular, a 50 Hz resampling with cubic interpolation was performed to ensure uniformity of the time baseline and avoid timestamp jitter due to a variable device framerate at around 30 FPS. A 10 Hz low-pass filter was then applied to the resampled data in order to exclude high frequency signal noise from the analysis. 

**Figure 2:**
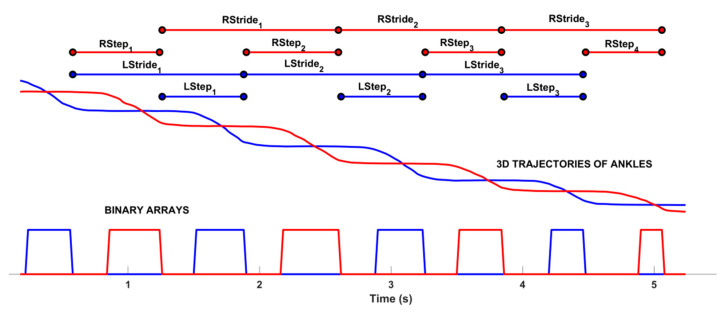
Results of the step segmentation algorithm: binary arrays for left (square line in blue) and right (square line in red) ankles estimated from 3D trajectories. At the top, some of the spatio-temporal parameters are shown; in particular, the step and gait cycle duration.

The second phase relied on a custom-written step segmentation algorithm explicitly designed to identify each step when the subject was inside the GAP zone. The step segmentation algorithm worked on the 3D trajectories of the ankle joints to avoid the loss of accuracy of foot joints [80]. When the subject entered the sensor’s field of view, the body-tracking algorithm started to provide the skeletal model. However, for the gait analysis with RGB-D system, it was essential to detect the time when the subject entered and left the GAP zone to identify the time window to be analyzed. To this end, the algorithm firstly estimated the 3D body center of mass (COM_BODY_) as in [51] and then used its 3D Euclidean distance from the sensor to determine the time when the subject entered and left the GAP, as in [72]. After identifying the time window, the step segmentation algorithm used the 3D trajectories of left (ANK_L_) and right (ANK_R_) ankles to analyze each leg individually inside the GAP and estimate the related gait parameters. In particular, the z-component of the 3D joint was used to apply a binary thresholding in order to identify the “stationary” and “in movement” ankle periods. The stationary ankle period was when the difference between two consecutive z-component values was less than the prefixed threshold; the “in movement” ankle period was when the difference was over the prefixed threshold. As in [72], the threshold was 2 cm: the aim was to verify that the threshold defined for post-stroke subjects was also valid for PD subjects and that the step segmentation algorithm worked exactly the same way without tuning parameters. The results of the step segmentation algorithm were two binary arrays that allowed the extraction of some traditional gait parameters per leg and overall, as in other reference studies using RGB-D approaches [37,46,56,61,66]. Figure 2 shows an example of the sequence of steps and some temporal parameters estimated from the binary arrays.

According to standard biomechanical protocols, the level walking analysis with 3D-GA commonly estimates many parameters, but only a subset is traditionally considered clinically relevant to highlight gait disorders [81]. For this reason, this study focused only on some spatio-temporal parameters that typically characterize gait patterns in post-stroke and PD subjects. It is important to note that the RGB-D and 3D-GA systems work on detecting different and specific events to estimate the same gait parameters, as in [72]: the two systems present differences in the physical location of passive markers and skeletal model joints, other than in the data processing algorithms. Nevertheless, starting from the events detected, it was possible to estimate the same spatial and temporal parameters and their average value inside the GAP, both for each side (i.e., step length, speed, time, and so on) and the overall walking test (i.e., cadence).

Another relevant feature of impaired gait pattern is related to the body sway during walking [82,83]: in fact, evident body sways are the consequence of the attempt to correctly balance walking in the presence of impairment and altered postural attitude while walking, and this is true both for post-stroke and PD subjects. For this investigation, the 3D-GA system estimated the position of the body center of mass from the 3D trajectories of the passive markers [84] and its peak-to-peak sways along the medio-lateral and vertical directions. Again, to limit the differences in locations of the body’s center of mass, the RGB-D system computed the 3D midpoint of HIP_L_-HIP_R_ segment (COM_HIP_): its peak-to-peak sways along the medio-lateral and vertical directions were computed and compared with the same parameters estimated by the 3D-GA system. Parameters estimated from the center of mass were relative to the overall walking test. 

### 2.5. Statistical Analysis

Three consistent trials were selected and considered for the analysis. For the estimation of the spatio-temporal parameters, the two body sides were analyzed separately, while the COM parameters were estimated as a single value for each trial.

After applying the Kolmogorov–Smirnov test, a non-parametric analysis was considered as the parameters were not normally distributed; thus, the median and quartile values of all the parameters were computed. The statistical analysis included two phases.

Firstly, all the statistical tests were performed on the entire group of patients (PS and PD subjects). The Wilcoxon test was used to compare the measurements obtained by the RGB-D system and those obtained by 3D-GA. The research for correlation between the two methods was performed using Spearman’s rank-order correlation, while, to assess the absolute agreement, the Intra Class Correlation (ICC) was used [85]. 

Then, the Bland–Altman plot was created to display the level of agreement between the RGB-D and the optoelectronic systems. This is a graphical method for comparing two measurements of the same variable in which the *x*-axis represents the mean of two measurements, and the *y*-axis represents the difference between. The plot can then highlight anomalies. For example, if one method always gives too high a result, then all points are above or below the zero line. It can also reveal that one method overestimates high values and underestimates low values. Otherwise, if the points on the Bland–Altman plot are scattered all over the place, above and below zero, then it suggests that there is no consistent bias of one approach versus the other. 

Then, the analysis was performed by considering the two groups of patients (PS and PD subjects) separately. A repeated measure ANOVA was performed on the parameters with the “within-subject” factor of systems (3D-GA vs. RGB-D system) and the “between-subject” factor of groups (PD vs. PS group). Post hoc tests were performed, where appropriate, for the significance threshold. A significance level of 0.05 was implemented throughout. The statistical analysis was performed using Minitab^®^ (version 18.1, State College, PA, USA).

## 3. Results

In this section, we present the results of our study, which were determined from the data collected on two groups of subjects during the experimental test. In particular, we enrolled eleven post-stroke subjects (average age: 53.3 ± 13.9 years; 3 females and 8 males; weight: 84.0 ± 21.7 kg; height: 1.8 ± 0.1 m; BMI: 25.6 ± 4.2 kg/m^2^), with the following characteristics: four subjects with left and seven with right hemiparesis; 4.36 ± 1.54 years from stroke event. Sensory deficits were present in nine patients, homonymous hemianopia in five patients, dysphasia in four patients, and visuospatial disorders in five patients. In addition, we enrolled ten PD subjects (average age: 66.4 ± 13.7 years; five females and five males; weight: 85.6 ± 15.4 kg; height: 1.7 ± 0.1 m; BMI: 30.1 ± 5.8 kg/m^2^) with the following features: 2.1 average H&Y score; 11.2 ± 7.5 years from disease diagnosis.

### 3.1. Statistical Analysis and Correlation Results

Firstly, the comparison results between the measurements obtained by the RGB-D system and those obtained by 3D-GA are reported in Table 1, where the median values, with the first and third quartiles, for each parameter, estimated on all participants’ walking performance (eleven PS and ten PD individuals) captured simultaneously by the two systems, are shown. The analysis indicated no statistical differences between the two systems’ groups (*p* ≥ 0.05), showing the agreement between the two systems. The only exception is related to the step width for which a statistical difference was found (*p* < 0.001). 

All the parameters indicated good reliability between the measurements (V sway showed moderate reliability), with the exclusion of step width, which displayed poor reliability (ICC = 0.44). The Spearman’s correlation values between measures from the 3D-GA and RGB-D systems were all statistically significant (*p* < 0.05) and generally good for the measurements; only step width and V sway showed moderate correlation values, confirming the results obtained for ICC.

These results indicate, in general, a good agreement between the two methods.

**Figure 3:**
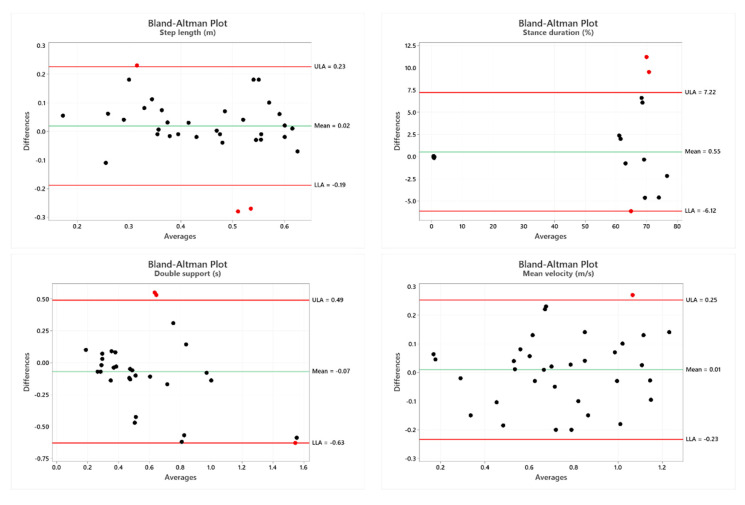

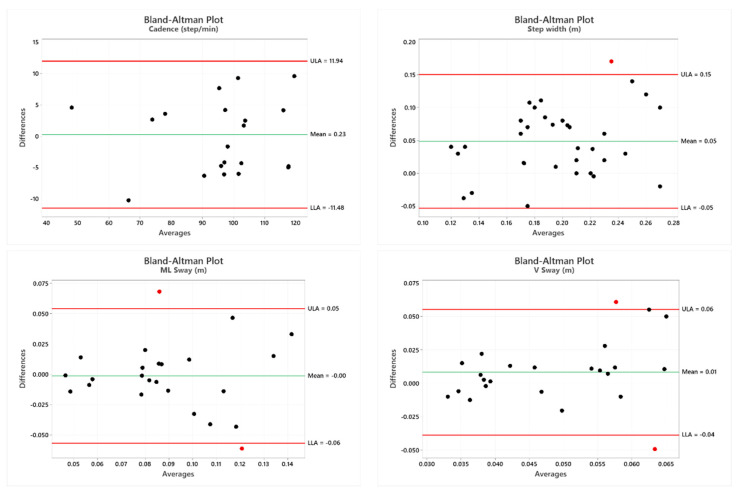
Bland–Altman plots of the mean of 3D-GA and the RGB-D systems against the difference between the two methods for the spatio-temporal parameters and the COM Sway.

In Figure 3, the Bland–Altman plot is displayed. It is a scatterplot of the mean of the RGB-D system and instrumented 3D-GA method plotted against the difference between the two methods [86]. It is possible to observe that the Bland–Altman graphs globally display a good agreement between the two measurement systems as most of the points fell within the interval. 

### 3.2. Gait Patterns in PD and PS Subjects

**Figure 4:**
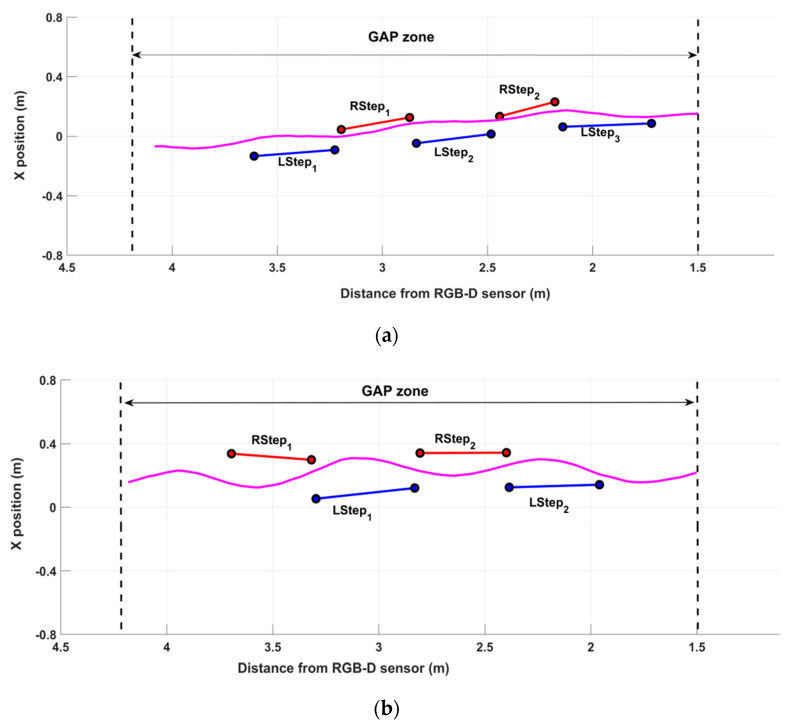
Gait patterns of one PD (**a**) and one post-stroke (**b**) participant. The black dotted lines delimit the GAP zone. The *x*-axis represents the 3D distance of the subject from the RGB-D sensor, while the *y*-axis represents the position relative to the sensor’s *x*-axis. The magenta lines represent the trajectory of COM_HIP_.

In order to demonstrate the ability of the system in the qualitative characterization of the walking performance, the following Figure 4 shows two examples of gait patterns. In particular, Figure 4a represents the walking pattern of one PD participant, while Figure 4b represents the walking pattern of one post-stroke (PS) participant. The aim of Figure 4 is not to highlight the differences between the walking scheme of the two pathologies but to verify the system’s ability to detect gait features in every circumstance, that is, in different subjects affected by distinct pathologies with their own peculiar gait characteristics. The PD subject (Figure 4a) performed five complete steps inside the GAP zone, whose length was short and variable on both sides: the first and the last steps were at the limits of the GAP zone, so the analysis procedure did not consider them. The graphical representation of the COM_HIP_ trajectory points out the small lateral sways during the walking test. This qualitative information suggests impairment during gait. On the contrary, the post-stroke subject (Figure 4b) performed four steps inside the GAP zone. The left steps seemed longer than the right ones, denoting a symmetry deterioration in the walking scheme. In addition, lateral sways seemed more accentuated than the PD subject, indicating greater instability and compensatory strategies during gait.

However, quantitative and objective measurements should support the qualitative evidence derived from the simple graphical representation. As reported in Table 2, the estimated spatio-temporal and COM parameters confirmed the preliminary qualitative indications. On average, PD performance showed an asymmetric gait, in length and speed, characterized by short steps (step length), slight slowness (mean velocity and cadence), and high stance phase (percentage of the gait cycles). In addition, the data confirmed the low lateral sways of the body’s center of mass. On the other hand, PS performance also showed asymmetry during walking, both in length and speed, but longer steps and higher slowness than PD performance, slightly greater than double support duration, and less stance duration. The data also confirmed more significant medio-lateral sway than PD performance. In contrast, the step width and vertical sway were comparable, confirming the lesser significance of these parameters. Finally, as expected, the best performance was relative to the left side in both cases, which was in line with the subjects’ conditions: the PS subject showed a right-side residual hemiparesis, while the PD subject exhibited more important symptoms on the right-side. 

The following radar graphs summarize the differences in the most significant spatio-temporal parameters between PD and PS performance. Since the gait parameters represent different physical quantities, they were normalized before representing them graphically. In particular, a minimum–maximum normalization was used by considering the estimated parameters for all the PD and PS walking tests that were collected and analyzed. Then, each parameter value was scaled to be more effectively represented in a range 0–1, using a minimum–maximum normalization. To this end, we considered the average normative values for healthy adult subjects [81] as the best walking performance, which practically would be associated with the maximum values (i.e., 1). On the contrary, the lowest parameter values estimated on the PD and PS groups were considered to represent the worst walking performance associated with the minimum values (i.e., 0). It is important to note that a direct relationship with the gait impairment characterized some parameters (stance duration, double support duration, and step width): in this case, the values of the parameters increased with increasing gait dysfunctions. Therefore, according to our choices regarding the graphical representation of the radar graphs, the worsening of these parameters (i.e., higher parameter values) corresponded to normalized values closer to zero.

**Figure 5:**
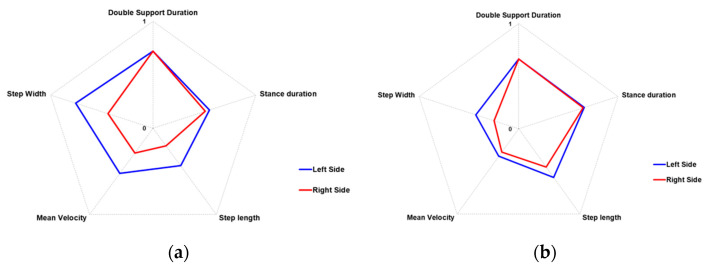
Radar charts of the relevant gait parameters for the left and right sides of the PD (**a**) and PS (**b**) performance.

Conversely, other parameters (step length, mean velocity) were characterized by an inverse relationship with the gait impairment: in this case, the values of the parameters decreased with increasing gait dysfunctions. The worsening of these parameters (i.e., lower parameter values) corresponded to normalized values closer to zero. In this way, all the normalized parameters were within the 0–1 range, producing radar charts that expanded outward when the gait performance was good, and collapsed inward when the gait performance was impaired. The radar graphs corresponding to Figure 4 and Table 2 are shown in Figure 5.

### 3.3. Analysis of the PD and PS Groups

The median and quartiles for each parameter are presented in Table 3 for each group of subjects and both systems. Measurements, commonly attributable to the left and right sides, were averaged for this analysis.

The results of the “between-subject” analysis of Group (that is, PD and PS groups), the “within-subject” analysis of System (that is, 3D-GA and RGB-D systems), and the Group × System interaction for each parameter are reported in Table 4. 

The analysis confirms that, in general, there were no significant differences between the two systems in the estimation of the spatio-temporal parameters and the sways of the center of mass. The only exception seemed to be related to step width, where slightly significant differences were highlighted both between groups and systems (but not in the Group × System interaction): this suggests that step width is probably a very challenging and sensible parameter related to the different positions of passive markers and joints of the skeletal model.

Regarding the gait cadence, the analysis showed a significant difference between groups (i.e., PD and PS) but not between systems (i.e., 3D-GA and RGB-D): the two systems seemed to agree in the estimation of the gait cadence; therefore, it could be an effective discriminatory parameter between the two groups of subjects. The same also occurred for ML sway, which showed a significant difference between groups (i.e., PD and PS) but not between systems (i.e., 3D-GA and RGB-D): again, this suggests that ML sway could be a valuable parameter to differentiate the two groups of subjects.

### 3.4. Comparison Versus Other Studies

Some studies in the literature have analyzed and validated technologies and methodologies against the “gold standard” 3D-GA. Nevertheless, the direct comparison of the results is generally problematic because not all studies consider the same spatio-temporal parameters, correlation methods, populations, or technological approaches. 

An approach based on a single RGB-D sensor was used in [37] on healthy young adults: this study reported a slightly greater agreement with 3D-GA than our findings, in particular for step length (ICC= 0.93 [37] vs. ICC = 0.81 [our]) and average velocity (ICC = 0.96 [37] vs. ICC = 0.95 [our]) under normal pace conditions. In [40], a multi-RGB-D sensors approach (four cameras along the 10-m walkway) was used on healthy subjects: in this study, the step width showed the lowest correlation (ICC = 0.65 [40]) compared to the other spatio-temporal parameters. The same occurred in our study, in which we obtained an even lower agreement value (ICC = 0.44 [our]).

Concerning the step width, our results for the Spearman correlation (r = 0.45 [our]) and agreement (ICC = 0.44 [our]) were comparable with the findings in [41], where values for the Pearson correlation (r = 0.52 [41]) and agreement (ICC = 0.40 [41]) were reported.

However, the latter results for step width seem to disagree with [43] that reports a high Pearson’s correlation coefficient (r = 0.85 [43]): in this study, the first model of Microsoft Kinect was used, combined with a treadmill, on healthy participants. Again, with respect to [43], our estimated Spearman correlation (r = 0.59 [our]) was globally lower than the Pearson correlation of stride time and stance time (r = 0.85 [43] and r = 0.77 [43], respectively). On the contrary, the result for double support seemed much better (r = 0.24 [43] vs. r = 0.71 [our]).

The same controversial results for step width are present in [66] both as agreement (ICC = 0.84 [66]) and Pearson’s correlation coefficient (r = 0.73 [66]): also, in this case, the first model of the Microsoft Kinect was used in conjunction with a treadmill on healthy participants. Regarding the other parameters, the results in [66] are worse for step length (ICC = 0.76 [66] vs. ICC = 0.81 [our]) and mediolateral sway (ICC = 0.84 [66] vs. ICC = 0.94 [our]), referring to the lowest speed setting on the treadmill.

In [36], two Kinect v.1 and an automatic algorithm for step segmentation were used to compare gait parameters on healthy children: the reported findings on average correlation and agreement are slightly greater than our results for step length (r = 0.79 [36] vs. r = 0.67 [our]; ICC = 0.85 [36] vs. ICC = 0.81 [our]), but lower for average velocity (r = 0.79 [36] vs. r = 0.90 [our]; ICC = 0.77 [36] vs. ICC = 0.95 [our]) and cadence (r = 0.79 [36] vs. r = 0.82 [our]; ICC = 0.79 [36] vs. ICC = 0.97 [our]).

Regarding the COM sway parameters during walking, objective results on correlation or agreement do not appear to be available; nevertheless, the high average sway along mediolateral direction suggested a concordance with the conclusions on pathological gaits in [83].

The direct comparison between parameter values is even more difficult because only a few validation studies specifically work on post-stroke and PD populations. Limiting the comparison to the spatio-temporal parameters of Table 3 and to the RGB-D sensor approaches, our average values were in line with the normative data (age 50–59 years) of post-stroke subjects in [61], as regards the step length (mean = 0.46 [61] vs. mean = 0.47 [our]), double support (mean = 0.47 [61] vs. mean = 0.52 [our]), and step width (mean = 0.21 [61] vs. mean = 0.19 [our]). On the contrary, the average values for walking speed (mean = 0.81 [61] vs. mean = 0.72 [our]) and cadence (mean = 94.34 [61] vs. mean = 89.52 [our]) were lower in our study, but this was probably related to the different composition of the PS group. For the same reason, the average value for step length was in line with [56] (mean = 0.51 [56] vs. mean = 0.47 [our]), while the walking speed (mean = 0.87 [56] vs. mean = 0.72 [our]) and double support (mean = 0.40 [56] vs. mean = 0.52 [our]) were slightly different.

Regarding studies on subjects with PD, our average values were comparable with those reported in [46] (H&Y 2.5, single task condition), in particular for cadence (mean = 98.8 [46] vs. mean = 101.81 [our]) and step width (mean = 0.10 [46] vs. mean = 0.15 [our]). However, there were some differences regarding double support (mean = 0.4 [46] vs. mean = 0.65 [our]) and walking speed (mean = 0.9 [46] vs. mean = 0.77 [our]), probably due to the different motor conditions of subjects included in our group. The average values were also directly comparable with [66] for cadence (mean = 101.16 [66]), walking speed (mean = 0.83 [66]), and stance duration (mean = 73.0% [66] vs. mean = 67.29% [our]).

The mean values of parameters were also comparable with studies that used different technological approaches, particularly inertial and wearable sensors, to analyze gait and compare spatio-temporal parameters with gold reference systems. For example, [68] and [12] employed inertial sensors and presented results on PD and post-stroke subjects. In [68], the mean values obtained for walking speed are greater than our findings, both for PD (mean = 0.85 [68] vs. mean = 0.77 [our]) and for post-stroke (mean = 0.61 [68] vs. mean = 0.72 [our]). On the contrary, the mean values obtained for stance duration are very similar, both for PD (mean = 66.70% [68] vs. mean = 67.29% [our]) and for post-stroke subjects (mean = 66.70% [68] vs. mean = 66.91% [our]). We also compared the mean values with [12]: in this case, the differences for stance duration were slightly higher than with the previous study. In particular, the most evident difference referred to the PD group (mean = 55.9% [12] vs. mean = 67.29% [our]) rather than post-stroke subjects (mean = 64.5% [12] vs. mean = 66.91% [our]): this probably depended on some differences in age, disease onset, and severity of the PD subjects involved.

However, despite some minor discrepancies due to different technological and methodological approaches in the referenced studies, the results globally demonstrated the reliability of the proposed solution and segmentation algorithm to estimate relevant spatio-temporal gait parameters that agree, concerning range and correlation, with studies that rely on different approaches and populations.

## 4. Discussion

In order to test whether an RGB-D sensor is capable of identifying gait characteristics and deviations during level walking in neurological diseases (in particular, post-stroke and PD), the walking abilities of two small cohorts of subjects were analyzed simultaneously with a single-camera solution and a 3D-GA system. In particular, the standard spatio-temporal parameters and the estimation of the center of mass excursion during gait were compared. The single-camera solution relies on an RGB-D sensor (i.e., Microsoft Kinect v2) and its body-tracking algorithm is able to track, in real-time, the 3D movements of the human body during gait on a reduced walking path (GAP) that is suitable for domestic environments. For the experimental procedure, the GAP was set along the traditional walking path used by the instrumented 3D-GA in order to allow a fair comparison and validation of the two systems. A custom-written step segmentation algorithm extracted some standard spatio-temporal parameters from the 3D trajectories of the skeletal model, provided by the optical sensor, by detecting every step performed inside the GAP. The computed COM information was used to determine when to perform the gait analysis inside the GAP (i.e., when the subject entered and left the GAP) and then to measure any dynamic balance anomalies during gait.

The results show that, using the implemented step segmentation algorithm, the RGB-D system provided an estimation of the same standard parameters measured with the optoelectronic system during the gait analysis test. Any slight differences between some measurements are probably attributable to the different reference points (i.e., the 3D position of markers on body and joints constituting the skeletal model) or to the different algorithms used by the two systems to estimate gait parameters. Consequently, this could have affected the agreement between the two systems and make it complex to compare results directly with other studies.

Despite this, our results exhibited a good agreement between the two systems in all the analyzed parameters, with the exception of the step width. No statistical differences were found between the two systems, and good reliability between the measurements was displayed according to the values of ICC. In addition, the research on the correlation between the 3D-GA and RGB-D systems exhibited values that were statistically significant, demonstrating, in general, a fair proportionality between the two methods. It is important to underline that, in general, the correlation values and ICC presented high and satisfactory values for all the parameters, with the exception of step width, as previously anticipated, and the V sway parameters. Unlike the other parameters, the results related to these two parameters showed, in fact, only moderate reliability (ICC = 0.44 for step width, ICC= 0.60 for V sway) and correlation values (r = 0.45 for step width, r = 0.48 for V sway) between the two measurements. The results were also confirmed by the Bland–Altman plots, which globally displayed a good association between the two measurement systems as most of the points fell within the defined interval.

The lower agreement obtained for the step width could depend on the close position of the two feet in some of the tests, which resulted in a lower precision in the estimation of the *x*-axis distance by the RGB-D system.

Similar considerations could be made for the V sway parameter. Although there were no particular concerns about this index, it is evident that the obtained results, in terms of ICC and correlation values, were lower than those of the other parameters. These differences could be related to the different 3D COM positions estimated by the two systems, which could have slightly interfered with the reliability of the results; or, more likely, to the lesser relevance of vertical swaying during walking, which involves less marked up–down body movements.

However, the results generally confirmed those obtained in previous studies carried out on post-stroke and parkinsonian subjects, even if it is difficult to directly compare our data with the literature.

The main difficulties for a fair comparison rely on the use of different correlation methods (ICC, Spearman’s correlation, and Pearson’s correlation), technologies (optical sensors, wearable sensors, and optoelectronic systems), experimental setup (single-camera, multi-camera, and treadmills), and populations (healthy, pathological subjects, adults, and young people): several studies in the literature adopt different methodological and technological approaches and analyze gait in different groups of subjects, so the comparison of results with other researches is not easy.

Concerning the agreement between vision-based and 3D-GA systems, as previously highlighted in Section 3.4, in [37], a slightly higher ICC agreement for mean velocity and step length was reported, while a greater Pearson’s correlation was estimated for cadence in [40]. On the contrary, our results showed a greater agreement for step length and mediolateral sway than [66]; and for step length, mean velocity, and cadence than [66]. The results for step width seem to be more controversial in the literature: for example, while [40,41,72] obtained the lowest correlation and agreement for step width as our analysis, other studies [43,66] presented satisfactory agreement data both in terms of ICC and Pearson’s correlation. The Spearman’s correlation for the Stance duration, expressed as a percentage of stride time, seemed to be in agreement with the Pearson’s correlation of stride time and stance time in [43], while the agreement seemed to be greater in our study for double support. The COM excursion results displayed high deviations, especially in the mediolateral direction, as expected in pathological gait patterns [83]. The weak results related to the vertical direction could be related to the different 3D COM positions (*y*-axis) considered by the two systems, as discussed and confirmed in our previous research [72].

Considering the PD and PS groups separately, the ranges of values estimated by the two systems were found to be in line, as evidenced by the median and the quartiles in Table 3, except for minor discrepancies due to the different landmarks and analysis algorithms. These data demonstrates that the RGB-D system is able to extract relevant features of gait with an accuracy comparable to the 3D-GA for both pathological conditions and this aspect is the most important finding of our study, according to its aim. The graphical representation of gait patterns and COM trajectories provides an immediate and intuitive qualitative indication about the global gait performance of PD and PS subjects (Figure 4): this is, however, supported by objective measures (Table 2) and by the summary representation of normalized parameters in the form of radar charts (Figure 5), which makes it possible to easily quantitatively compare performance over time, to reveal differences between body sides (as in Table 2, where more impairment is evidenced for the right side), and to highlight different gait strategies for PD and PS subjects (as in Table 2, where there are relevant dissimilarities in mean velocity, cadence and ML sway between the two evaluated subjects).

Specifically, the overall comparison between PS and PD groups confirmed that the two systems did not show significant differences in estimating the spatio-temporal parameters and the sways of the center of mass, as highlighted by the System and Group × System analysis (Table 4).

The data analysis showed slightly significant differences in step width between populations and systems. Furthermore, the same data were found to not be relevant for the Group × System interaction: this confirmed that step width is probably a challenging parameter to compare due to the different placement of passive markers and skeletal model joints.

Regarding the gait cadence, there were significant differences between groups (i.e., PD and PS) but not between systems (i.e., 3D-GA and RGB-D): this suggests that the two systems agreed in the estimation of the gait cadence, which could, therefore, be an effectively discriminating parameter for different gait schemes due to different neurological impairments. The analysis also showed a significant difference between groups (i.e., PD and PS) but not between systems (i.e., 3D-GA and RGB-D) concerning ML sway: this suggested that the two systems agreed in the estimation of ML sway that could be a valuable parameter to differentiate the two populations. It is, in fact, highly probable that the gait impairment due to hemiplegia in post-stroke subjects is associated with a more dangling walking to compensate for gait imbalance.

According to the overall analysis, the RGB-D system seemed able to extract important features of the gait patterns, in line with the 3D-GA system and regardless of the complexity of gait of the two neurological diseases considered: this suggested the suitability of the RGB-D system to perform objective measures of walking strategies where 3D-GA is not applicable, such as domestic environments.

Particularly relevant is the fact that the obtained measures were found to be comparable with those obtained by means of the optoelectronic system, which represents the gold standard for movement analysis.

These results are also significant from a clinical and rehabilitative point of view. The monitoring of gait parameters using an easy and non-invasive testing system may be helpful in clinical settings to expand the number of patients examined. The RGB-D system could be considered a valid means for a preliminary, quick, easy, and low-cost evaluation of gait variables. However, it is important to underline that the proposed system does not intend to replace gait laboratories based on optoelectronic systems. The optoelectronic systems should continue to be used in clinical settings for a more in-depth assessment of gait strategies and clinical decision-making. On the other hand, the more relevant advantages of the RGB-D solution could be in unsupervised settings, overcoming the limitations of the traditional gait analysis in laboratory settings and—not of secondary importance—without undressing patients for marker placement, which is frequently a psychological barrier for frail patients [21,23]. Furthermore, outpatient rehabilitation facilities could benefit from the information obtained from the proposed system, which could provide quantitative gait parameters even outside of gait-analysis laboratories, without excessively interfering with the subject’s usual activities.

For rehabilitation purposes, the application of these kinds of solutions could be exploited both in the hospital (at entrance and discharge of patients), in the outpatient ambulatory, and in-home settings. In addition to objective evaluation of gait patterns, solutions based on optical devices, such as the RGB-D sensors, could be helpful also for remote rehabilitation, which has proved to be a promising intervention to reduce the effect of impairment and improve quality of life both for post-stroke and parkinsonian subjects.

In both pathologies, continuous rehabilitation and physical activity are required to reduce motor and cognitive decadence. The possibility of simple clinical monitoring would allow a more specific and targeted therapeutic action in wider cohorts of patients.

Several systematic reviews [87,88,89,90] agree on the effectiveness of remote rehabilitation interventions for recovery from the motor, cognitive, and psychological dysfunctions linked to neurological events. This aspect could be crucial, especially in countries with a scarcity of socio-economic resources. In addition, the recent COVID pandemic restrictions have also made it clear that telemedicine could become a valid and desirable means to continue health care at home, especially in frail subjects, even in the advanced economies. The current pandemic scenario has highlighted the need to offer telemedicine, remote assistance, and monitoring services when access to health facilities is limited or impossible. In this way, it would be possible to follow patients remotely, especially in cases of chronic and disabling diseases, avoiding the worsening of psycho-physical conditions and the onset of a sense of abandonment, as recent studies on the effects of the pandemic have shown. Several systems are now available for the remote monitoring of subjects with specific pathologies in the real world, including diabetes [91], osteoarthritis [92], respiratory dysfunctions [93], amyotrophic lateral sclerosis [94], and cognitive rehabilitation [95].

In this context, the system we described could permit the real-time integration of remote objective motor evaluation with exergames and ecological exercises, allowing more intensive rehabilitation training activities to be carried out at home with lower costs. This possibility represents a strength of the system: the specialists in the rehabilitation sector, in fact, claim greater effectiveness for the exercises defined as “ecological”, that is, referring to the behaviors, habits, and activities of daily life. This result could be achieved through easy-to-use and low-cost solutions, such as the one we proposed, that constantly and intensively analyze, monitor, evaluate, stimulate, and rehabilitate the patients, developing their motor and cognitive potential to the maximum.

Nevertheless, the study has some limitations. At present, the small sample size could limit the generalization of the results to a broader range of disease severity and other neurological pathologies. In subsequent studies, we will extend the sample size by including more PD and PS subjects with wider disability scores to confirm our preliminary results, and we are planning to also collect data in-home settings. In addition, the following point must be made regarding the use of Microsoft Kinect© v2 as the RGB-D sensor of the proposed solution: although the device was discontinued a few years ago, it is still widely used for clinical research, as evidenced by several recent studies in the literature. In any case, the availability on the market of other RGB-D sensors will allow us to improve the current analysis and results using non-invasive technologies and vision-based approaches with higher performance and accuracy, as recent studies have shown [96,97].

It is important to underline that, in this study, only the spatio-temporal parameters and the COM sways were analyzed, even if other kinematic variables were calculated. However, it is well-known that spatio-temporal parameters are among the measurements most used by clinicians for evaluating gait patterns in post-stroke and parkinsonian patients. Effectively, the spatio-temporal parameters accurately represent the patient’s ability to satisfy the general gait requirements, such as weight acceptance, support on a single limb, and progression of the swing [98]. They are also able to highlight the presence of asymmetrical gait strategies, due, for example, to anomalous stance phase, double support duration, slow walking speed, or shorter steps: these are among the main features considered in the clinical practice for the characterization of pathological gait patterns on which to evaluate, for example, the effects and benefits of rehabilitation treatments. By considering only reliable measurements, we can still obtain a significant clinical picture of the gait. In future studies, other relevant information on walking strategies could be analyzed more in-depth, such as upper limb movements and postural attitude during gait, thereby improving the overall characterization of pathological gait and focusing on the specific neurological condition of interest.

In conclusion, it is important to underline that even if this study focuses only on gait analysis, one of the most challenging motor tasks for vision-based solutions, it is part of a broader project on parkinsonian and post-stroke subjects. The project aims, in fact, to automate and comprehensively assess the motor conditions of pathological subjects through RGB-D sensors and computer vision techniques, thus adopting non-invasive and easy-to-use approaches that are suitable for unsupervised contexts, such as the home setting.

## 5. Conclusions

This study proposed a vision-based solution for assessing gait patterns and dysfunctions due to neurological impairment associated with post-stroke and Parkinson’s disease comorbidities by means of a single RGB-D sensor capable of capturing the 3D trajectories of body movements on a reduced walking path that is also suitable for domestic settings.

The qualitative analysis of the spatio-temporal parameters and COM demonstrated the capability of the RGB-D system to extract gait characteristics and differentiate specific gait patterns in agreement with the more complex and demanding gold standard 3D-GA. In fact, the data analysis confirmed that no difference between the two systems was statistically significant, except for step width, the results of which were in line with other reference studies: this indicates that the two systems measure the same quantities. It is noteworthy that some parameters, particularly cadence and ML sway, showed statistically significant differences between the two groups of subjects, suggesting that the system is also able to catch discriminatory parameters in populations with different gait patterns. However, a more in-depth analysis is necessary to confirm this finding by enlarging the sample size.

The ability to estimate gait parameters in agreement with standard 3D-GA systems is essential to monitor gait parameters in unsupervised settings, such as domestic environments, where traditional 3D-GA systems are not applicable. At this stage of development, immediate clinical implications relate to the possibility to schedule rehabilitation programs at home, both exclusively or in combination with hospitalization periods. In fact, the non-invasiveness, portability, and easy-to-use features of the proposed solution allow the combining of evaluation and rehabilitation tasks for home settings, thus defining new strategies for the management of neurological diseases and frail subjects. Future developments could be focused to the potential diagnostic capability of the system: in this case, the high sensibility of the system for detecting minimal alterations of movements could be exploited as a support for clinical judgments and differential diagnoses.

## Figures and Tables

**Table 1:** Median (first and third quartile) values for spatio-temporal and COM parameters estimated for the two systems, ICC and values of Spearman’s correlation coefficient between the 3D-GA and RGB-D system (*: *p* < 0.05).

**Spatio-Temporal** **Parameters (Unit)**	**3D-GA System**	**RGB-D System**	**ICC**	**Spearman’s Correlation**
Step length (m)	0.43 (0.37, 0.56)	0.45 (0.32, 0.56)	0.81	0.67 *
Stance duration (%)	66.50 (62.65, 71.60)	67.00 (63.35, 71.19)	0.78	0.59 *
Double support duration (s)	0.46 (0.31, 0.91)	0.53 (0.34, 0.77)	0.80	0.71 *
Mean velocity (m/s)	0.74 (0.59, 0.99)	0.76 (0.54, 0.95)	0.95	0.90 *
Cadence (step/min)	97.20 (85.50, 105.18)	98.36 (87.77, 102.99)	0.97	0.82 *
Step width (m)	0.23 (0.19, 0.24)	0.17 (0.14, 0.20) *	0.44	0.45 *
**Center of Mass** **Parameters (Unit)**	**3D-GA System**	**RGB-D System**	**ICC**	**Spearman’s Correlation**
ML sway (m)	0.09 (0.07, 0.10)	0.09 (0.06, 0.12)	0.94	0.61 *
V sway (m)	0.05 (0.04, 0.06)	0.04 (0.04, 0.05)	0.60	0.48 *

**Table 2:** Spatio-temporal and COM parameters for the left and right sides of PD and PS performance shown in Figure 4.

	PD #4–Figure 4a		PS #7–Figure 4b	
Parameters	Left Side	Right Side	Left Side	Right Side
Step length (m)	0.39	0.29	0.45	0.39
Stance duration (%)	76.20	77.69	72.20	72.65
Double support duration (s)	0.60	0.60	0.70	0.70
Mean velocity (m/s)	0.77	0.56	0.59	0.55
Step width (m)	0.13	0.19	0.19	0.23
Cadence (steps/min)	114.65		79.47	
ML Sway (m)	0.05		0.10	
V Sway (m)	0.03		0.04	

**Table 3:** Median (first and third quartile) values for spatio-temporal and COM parameters estimated for the two systems of the PD and PS group.

	RGB-D System		3D-GA System	
Parameters	PD Group	PS Group	PD Group	PS Group
Step length (m)	0.36 (0.29, 0.58)	0.46 (0.39, 0.56)	0.39 (0.37, 0.42)	0.49 (0.38, 0.64)
Stance duration (%)	65.75 (63.67, 71.14)	67.50 (62.75, 71.75)	70.30 (62.55, 74.53)	65.00 (62.52, 69.50)
Double support duration (s)	0.66 (0.37, 0.77)	0.51 (0.31, 0.69)	0.73 (0.28, 0.91)	0.42 (0.33, 0.57)
Mean velocity (m/s)	0.61 (0.54, 1.14)	0.80 (0.54, 0–94)	0.71 (0.54, 1.11)	0.74 (0.62, 0.94)
Step width (m)	0.15 (0.12, 0.16)	0.19 (0.16, 0.22)	0.23 (0.14, 0.24)	0.24 (0.21, 0.26)
Cadence (steps/min)	103.55 (98.90, 113.92)	95.24 (72.58, 99.17)	102.20 (97.20, 118.00)	93.90 (75.20, 99.40)
ML Sway (m)	0.08 (0.06, 0.11)	0.09 (0.08, 0.12)	0.08 (0.06, 0.09)	0.10 (0.08, 0.14)
V Sway (m)	0.04 (0.04, 0.07)	0.05 (0.04, 0.09)	0.05 (0.04, 0.08)	0.04 (0.03, 0.04)

**Table 4:** Results of the “between-subject” analysis of Group (PD and PS groups), the “within-subject” analysis of System (3D-GA and RGB-D systems), and the Group × System interaction for spatio-temporal and COM parameters. Statistically significant data are highlighted in bold.

Parameter	Factor	F	*p*-Value	Partial η^2^
Step length (m)	Group	2.20	0.143	0.0333
	System	0.26	0.613	0.0040
	Group × System	0.03	0.869	0.0004
Stance duration (%)	Group	0.66	0.090	0.0995
	System	0.91	0.344	0.0140
	Group × System	0.96	0.332	0.0147
Double support duration (s)	Group	0.61	0.438	0.0094
	System	0.41	0.525	0.0063
	Group × System	0.18	0.677	0.0027
Mean velocity (m/s)	Group	0.45	0.502	0.0071
	System	0.01	0.903	0.0002
	Group × System	0.01	0.981	0.00001
Step width (m)	**Group**	**10.75**	**0.002**	**0.1438**
	**System**	**17.96**	**<0.001**	**0.2192**
	Group × System	0.001	0.982	0.0001
Cadence (steps/min)	**Group**	**9.50**	**0.003**	**0.1293**
	System	0.02	0.885	0.0003
	Group × System	0.10	0.752	0.0016
ML Sway (m)	**Group**	**6.17**	**0.017**	**0.1281**
	System	0.05	0.819	0.0013
	Group × System	0.40	0.533	0.0093
V Sway (m)	Group	0	0.958	0.0001
	System	0.05	0.824	0.0012
	Group × System	3.15	0.083	0.0698

## Data Availability

Data available on request due to restrictions e.g., privacy or ethical.
